# *Arabidopsis AtDjA3* Null Mutant Shows Increased Sensitivity to Abscisic Acid, Salt, and Osmotic Stress in Germination and Post-germination Stages

**DOI:** 10.3389/fpls.2016.00220

**Published:** 2016-02-25

**Authors:** Silvia Salas-Muñoz, Aída A. Rodríguez-Hernández, Maria A. Ortega-Amaro, Fatima B. Salazar-Badillo, Juan F. Jiménez-Bremont

**Affiliations:** Laboratorio de Biotecnología Molecular de Plantas, División de Biología Molecular, Instituto Potosino de Investigación Científica y TecnológicaSan Luis Potosí, México

**Keywords:** *Arabidopsis thaliana*, *AtDjA3*, abscisic acid, abiotic stress, heat shock proteins, J-protein

## Abstract

DnaJ proteins are essential co-chaperones involved in abiotic and biotic stress responses. *Arabidopsis AtDjA3* gene encodes a molecular co-chaperone of 420 amino acids, which belongs to the J-protein family. In this study, we report the functional characterization of the *AtDjA3* gene using the *Arabidopsis* knockout line designated *j3* and the *35S::AtDjA3* overexpression lines. Loss of *AtDjA3* function was associated with small seed production. In fact, *j3* mutant seeds showed a reduction of 24% in seed weight compared to Col-0 seeds. Expression analysis showed that the *AtDjA3* gene was modulated in response to NaCl, glucose, and abscisic acid (ABA). The *j3* line had increased sensitivity to NaCl and glucose treatments in the germination and cotyledon development in comparison to parental Col-0. Furthermore, the *j3* mutant line exhibited higher ABA sensitivity in comparison to parental Col-0 and *35S::AtDjA3* overexpression lines. In addition, we examined the expression of *ABI3* gene, which is a central regulator in ABA signaling, in *j3* mutant and *35S::AtDjA3* overexpression lines. Under 5 μM ABA treatment at 24 h, *j3* mutant seedlings displayed higher *ABI3* expression, whereas in *35S::AtDjA3* overexpression lines, *ABI3* gene expression was repressed. Taken together, these results demonstrate that the *AtDjA3* gene is involved in seed development and abiotic stress tolerance.

## Introduction

Seed germination and seedling establishment are the most critical stages of survival during the life cycle of an individual plant (Daszkowska-Golec, 2011). Seeds are exposed to a wide range of unfavorable environmental conditions that induce stress, and therefore have a negative impact on germination, growth, and development (Rao et al., 2006). As a result, seeds have developed defense mechanisms that allow them to tolerate and respond rapidly to unfavorable conditions (Koornneef et al., 2002; Vallejo et al., 2010).

Heat Shock Proteins (HSP) are accumulated during abiotic stress as a defense mechanism, and at the later stages of seed development appear to play a protective role in desiccation tolerance (Wehmeyer and Vierling, 2000; Koornneef et al., 2002). HSPs are involved in a variety of cellular processes including protein folding, assembly of oligomeric proteins, transport of proteins across membranes, stabilization of polypeptide strands and membranes, and prevention of protein inactivation (Vierling, 1991; Wang et al., 2004; Xue et al., 2010). In plants, HSPs are classified into five classes according to their molecular weight: HSP100, HSP90, HSP70, HSP60, and small HSP (sHSP; Wang et al., 2004).

HSP40 proteins, also referred to as DnaJ or J-proteins, are co-chaperones of the HSP70 machine. J-proteins are key players in stimulating HSP70 ATPase activity, thereby stabilizing its interaction with client proteins (Bukau and Horwich, 1998; Walsh et al., 2004). The J-proteins contain in the N-terminus region a highly conserved domain of approximately 70 amino acids, known as the J domain. This domain consists of four α-helices comprising two short helices (I and IV) and two tightly packed anti-parallel helices (II and III) linked by a loop region that contains a highly conserved tri-peptide (HPD: histidine-proline-aspartic acid), which is required for interaction with HSP70 proteins (Wall et al., 1994; Rajan and D’Silva, 2009). Adjacent to J domain is a characteristic glycine and phenylalanine (G/F) rich region. It has been proposed that this region serves as a flexible linker region and controls the specificity of J-protein functions (Craig et al., 2006). After the G/F-rich region there is a cysteine-rich region which forms a type I zinc-finger domain, which contains four repeated motifs (CXXCXGXG). This domain is essential for binding to unfolded protein and assists HSP70 with protein folding (Bukau and Horwich, 1998; Lu and Cyr, 1998). Finally, the C-terminal region, which is less conserved, is important for providing specificity for HSP70 J-protein machinery (Shi et al., 2005). Classification of J-proteins include the type I or A proteins, which present all the characteristic domains or regions; type II or B proteins, that lack the zinc-finger domain, and type III or C proteins that only contain domain J (Rajan and D’Silva, 2009). In the *Arabidopsis thaliana* genome, 116 J-proteins and four J-like proteins have been identified; of which eight belong to type I, 16 to type II, and 92 to type III (Rajan and D’Silva, 2009). The *A. thaliana* AtDjA3 protein belongs to the type I classification. In plants, J-proteins are induced under different stress conditions. *AtDjA3* gene is expressed in roots, stems, leaves, flower buds, flowers, and siliques, and its expression can be induced by heat, cold, and drought stress (Li et al., 2005, 2007), and also under saline conditions with alkaline pH (Yang et al., 2010).

In the present study, we deepen our understanding of *AtDjA3* gene under salt and osmotic stress, and the application of phytohormone abscisic acid (ABA). Expression analysis of the *AtDjA3* gene in *Arabidopsis* seedlings revealed that its expression is modulated by NaCl, glucose, and ABA. For the molecular characterization of *AtDjA3* gene, we analyzed the *Atdja3*-null (*j3*) mutant and *35S::AtDjA3* overexpression lines. Our results reveal that *j3* loss-of-function mutant produces small seeds that are less tolerance to salt and osmotic stress, reflected by a reduced germination rate and lower percentage of green cotyledons in comparison to the Col-0 and *35S::AtDjA3* overexpression lines. In addition, the *j3* mutant line shows more sensitivity to exogenous ABA. Our results suggest that *AtDjA3* gene plays a role in abiotic stress tolerance.

## Materials and Methods

### Plant Material and Growth Conditions

The *Atdja3* mutant line (*j3*) and transgenic lines (*OvJ3*-8 and -14) used in this study were generated in the *Arabidopsis thaliana* ecotype Columbia 0 (Col-0) background. The T-DNA insertion line (Salk_132923) for the *AtDjA3* gene (At3g44110) was obtained from the Salk Institute Genomic Analysis Laboratory^1^ (Alonso et al., 2003). The seeds used for all experiments were harvested at the same time. The seeds of *A*. *thaliana* Col-0, *Atdja3*-null mutant line (*j3*) and *35S::AtDjA3* overexpression lines (*OvJ3*) were sterilized with 20% (v/v) commercial sodium hypochlorite (6% free chlorine) solution for 5 min, and rinsed five times in sterile distilled water. Aseptic seeds were germinated and grown on agar plates containing Murashige and Skoog (MS) 0.5x medium supplemented with 7 g/L phytagel, and 1.5% sucrose (Murashige and Skoog, 1962). Plates were kept at 4°C for 3 days, and then incubated at 22 ± 2°C for 10 days in a growth chamber under a 16 h light (13,000 luxes)/8 h dark photoperiod. Plants were grown to maturity in soil pots, with a mixture of Sunshine Mix #3 commercial substrate, perlite and vermiculite (3:1:1), in a growth chamber at 22 ± 2°C with a 16 h light (13,000 luxes)/8 h dark photoperiod.

### Nucleic Acids Isolation and cDNA Synthesis

Genomic DNA was isolated from *A. thaliana* WT (Col-0) and T-DNA insertion mutant line plants using the method described by Murray and Thompson (1980). RNA extraction from Col-0, *Atdja3*-null mutant line and *35S::AtDjA3* overexpression lines was performed using the Concert Plant RNA Reagent (Invitrogen, Carlsbad, CA, USA) by following the manufacturer’s instructions; samples were stored at -70°C until analysis. For the removal of contaminating genomic DNA, RNA samples were treated with DNase I (Invitrogen, Carlsbad, CA, USA). Synthesis of cDNA was carried out with the Super Script II Reverse Transcriptase enzyme (Invitrogen, Carlsbad, CA, USA) according to the manufacturer’s instructions. The cDNAs were stored at -20°C for subsequent use.

### Identification of the T-DNA Insertional Mutant Line

Seeds of T-DNA insertion mutant line (Salk_132923) were germinated in plates containing MS 0.5x medium; after 10 days they were transferred to soil pots. For genotype analysis PCR assays were performed on genomic DNA isolated from 3-week-old plants using the T-DNA left border (LB) oligonucleotide and gene-specific oligonucleotides designed flanking the T-DNA. The oligonucleotides used were: FwSalk_132923 5′-CTTGAAGGTATCTCTTGAGGATGTGTACC-3′, RvSalk_132923 5′-GACGATGCATCTGAATACGTACCAGG-3′, and FwLB 5′- AGCAAGCGGTCCACGCTGGTTT-3′.

### Generation of *A. thaliana 35S::AtDjA3* Overexpression Lines

The *AtDjA3* open reading frame (At3g44110 GenBank ID: 823531) was amplified from a cDNA sample of *Arabidopsis* seedlings using the Hot Start High-Fidelity Polymerase Kit (Qiagen, USA), and the following oligonucleotides: FwAtDjA3 5′-GGCGAAAAGATGTTCGGTAGAGG-3′ and RvAtDjA3 5′-GTCTCTCTAAGGAGTTACTTACTGC-3′. The amplified product of 1,263 bp was cloned into the pCR8/GW/TOPO vector (Invitrogen, Carlsbad, CA, USA). The cloned products were sequenced using the M13 oligonucleotide in an ABIPRISM 377 DNA automated sequencer (Perkin Elmer, USA). The sequenced entry clone was recombined into the pMDC32 destination vector (Curtis and Grossniklaus, 2003), by site-specific recombination using the Gateway LR Clonase II Enzyme Mix (Invitrogen, Carlsbad, CA, USA). The pMDC32-*AtDjA3* vector was transferred into the *Agrobacterium tumefaciens* strain GV2260 by electroporation, and transformed into *Arabidopsis* plants WT (Col-0) by the “floral dip” method (Zhang et al., 2006). Transgenic lines carrying the *AtDjA3* gene (*35S::AtDjA3*) were selected on MS 0.5x medium containing 50 mg/mL hygromycin. To verify the expression levels of the *AtDjA3* gene in the two transgenic lines, RT-PCR analysis was carried out. For that, we used the following oligonucleotides: FwAtDjA3 5′-TGACGATGAAGATGATGACCATC-3′ and RvT-NOS 5′-ATTGCCAAATGTTTGAACGATCG-3′. As loading control, the *A. thaliana Actin8* (At1g49240) transcript was amplified using the FwAct8 5′- GCCAGTGGTCGTACAACCG-3′ and RvAct8 5′-CACGACCAGCAAGGTCGAGACG-3′ oligonucleotides. The T2 generation of transgenic plants was transferred into soil pots and grown in growth chambers under controlled conditions to produce seeds. Homozygous transgenic lines (T3) were used for the subsequent analysis of seed germination and in the stress tolerance assays.

### Quantitative RT-PCR (qRT-PCR) of *AtDjA3* Gene Under Abiotic Stress

Total RNA from *A. thaliana* was obtained from seedlings as described above and used for qRT-PCR assays. Possible genomic DNA contamination was removed using DNase I (Invitrogen, Carlsbad, CA, USA). RNA concentration was measured in a NanoDrop ND-1000 UV-Vis spectrophotometer (NanoDrop Technologies) before and after treatment with DNase I. cDNA synthesis and qRT-PCR analysis were performed by the one-step assay using the Power SYBR^®^ Green RNA-to-CT^TM^ One-Step Kit (Applied Biosystems, USA). The expression levels of the *AtDjA3* gene under salt, osmotic, and exogenous ABA treatments were assessed in 15-day-old *A. thaliana* Col-0 plants. Seedlings were transferred to liquid MS 0.5x medium supplemented with NaCl (150 and 175 mM), glucose (5 and 6%), and ABA (1, 3, and 5 μM) for 12 and 24 h. Expression level analyses of the *AtDjA3* gene were performed using the following oligonucleotides: FwqRTJ3n 5′-TGACGATGAAGATGATGACCATC-3′ and RvqRTJ3n 5′-GCAAGAGACAAATTGGTTGGAG-3′ for the *AtDjA3* gene (At3g44110), and UBQ5-F 5′-TCGACGCTTCATCTCGTCCT-3′ and UBQ5-R 5′-CGCTGAACCTTTCCAGATCC-3′ for the *UBQ5* (At3g62250) control gene. The expression level of the *ABI3* gene was measured using RNAs obtained from 15-day-old Col-0, *Atdja3*-null mutant and *35S::AtDjA3*-8 overexpression lines that were previously treated with ABA (0 and 5 μM) for 12 and 24 h. Expression level analyses of the *ABI3* gene were performed using the following oligonucleotides: FwqABI3 5′-CACAGCCAGAGTTCCTTCCTT-3′ and RvqABI3 5′-ATGTGGCATGGGACCAGACT-3′ for the *ABI3* gene (At3g24650), and FwAPT1 5′-GTCATCCCCGACTTCCCTAA-3′ and RvAPT1 5′-AGGCATATCTGTTGTTGCAGGT-3′ for the *APT1* (At1g27450) control gene. cDNA synthesis and quantitative PCR analyses were done in a 10 μL reaction mixture containing 50 ng of total RNA as template using the Power SYBR^®^ Green RNA-to-CT^TM^ 1-Step Kit (Applied Biosystems) as described previously (Salas-Muñoz et al., 2012; Rodríguez-Hernández et al., 2014). For each sample, three biological replicates were analyzed with their respective technical replicates. Experiments were repeated at least twice and gave similar results.

### Germination Assays Under Abiotic Stress and Hormone Treatments

Seeds of *A. thaliana* ecotype Col-0, *Atdja3*-null mutant line (*j3)* and *35S::AtDjA3* overexpression lines (*OvJ3*; T3) were germinated under different stress conditions. The effect of salt stress on germination was evaluated on MS 0.5x medium supplemented with 0, 125, and 150 mM NaCl. The effect of osmotic stress on germination was assessed on MS 0.5x medium without sucrose and supplemented with 0, 4, and 5% glucose. In addition, seeds were germinated in presence of different concentrations of ABA (0, 1, 3, and 5 μM). The ABA stock solution was prepared by dissolving ABA in small aliquots of 1N NaOH. The ABA stock was diluted with distilled water. The germination assays were carried out using 20 seeds per treatment. The seeds were germinated and grown vertically on petri dishes, and counted when the radicle emerged from the seed coat. In addition, the green cotyledon number was scored after 21 days of NaCl, glucose, or ABA treatments. Data are mean ± SE (*n* = 20) from five biological replicates. Experiments were repeated at least three times and gave similar results.

### Seed Weight Estimation of Col-0, *Atdja3*-mutant, and *35S::AtDjA3* Over-Expression Lines

Seed weight was calculated from three replicates for each line (*n* = 3), where 500 seeds represent each replicate. Each seed lot (1-month post-harvest) was measured on an analytical scale, and weights are expressed in milligrams. Experiments were repeated at least three times and gave similar results.

### Microscopic Analysis by Environmental Scanning Electron Microscopy

For environmental scanning electron microscopy (eSEM) analysis, dried seeds were glued onto pure carbon-containing polymer films, and then fixed onto eSEM sample holders. The external seed morphology of Col-0, *Atdja3*-null mutant line (*j3*), and *35S::AtDjA3* overexpression lines (*OvJ3*) were evaluated. The seed width and length were measured with a high-resolution scanning electron microscope (eSEM/QUANTA 200 FEI, Low Vacuum/Water). Morphological seed assays, including width and length of seeds, were carried out using 10 seeds of each genotype. Photomicrographs were obtained with the eSEM in a pressure chamber at 90–100 Pa and voltages of 15.0 and 30.0 kV.

### Statistical Analysis

To explore potential differences in germination, and green cotyledons among treatments for WT (Col-0), mutant line (*j3*) and transgenic lines (*35S::AtDjA3*-8 and -14), we used One-way ANOVA analysis through Tukey’s multiple comparison post-test using GraphPad Software. The data are presented as the mean ± standard error. Differences at *p* ≤ *0.05* were considered significant.

## Results

### Seed Morphology of *Atdja3*-Null Mutant Line (*j3*) and *35S::AtDjA3* Overexpression Lines (*OvJ3*)

**FIGURE 1:**
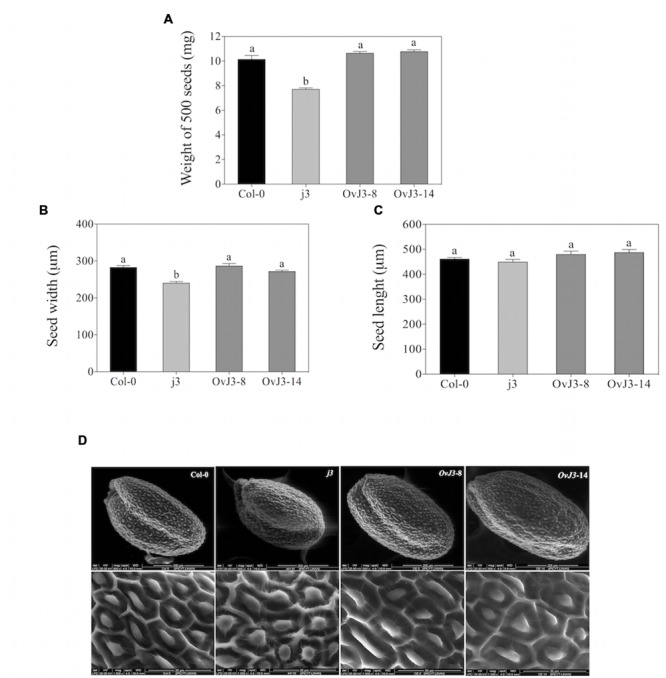
**Seed morphology of *Atdja3-*null mutant line (*j3*) and *35S::AtDjA3* overexpression lines (*OvJ3*-8 and -14). (A)** Weight of seeds (mg) from the Col-0, *j3* mutant line, and *35S::AtDjA3* overexpression lines. Data show means ± SE from the three groups of 500 dry seeds of each genotype. **(B)** Width and **(C)** Length of seeds (μm) from the Col-0, *j3* mutant line, and *35S::AtDjA3* overexpression lines. **(D)** Scanning electron micrographs showing: whole seed, scale bar = 200 μm; seed coat, scale bar = 50 μm; of Col-0, *j3* mutant line, and *35S::AtDjA3* overexpression lines. Error bars represent the means ± SE (*n* = 10). Different letters indicate significant differences between the Col-0, *Atdja3-*null mutant line, and *35S::AtDjA3* overexpression lines. One-way ANOVA analyzed significant differences among seeds lines through a Tukey’s test (*p* ≤ 0.05).

To address the biological functions of *AtDjA3* gene in seed morphology and response to abiotic stress, mutant, and overexpression lines were characterized. We selected the Salk_132923 line from the Salk T-DNA collection (Alonso et al., 2003), which contains a T-DNA insertion in the fourth exon of *AtDjA3* gene (Supplementary Figure S1A). The T-DNA homozygous line was identified by PCR, and the absence of the *AtDjA3* transcript was verified by RT-PCR (Supplementary Figure S1B), confirming that the Salk_132923 line is a null allele (*j3*) of the *AtDjA3* gene. In addition, we generated several transgenic *Arabidopsis* plants that overexpress the *AtDjA3* gene under the control of the CaMV 2X35S promoter (Supplementary Figure S1C). The expression levels of two independent *AtDjA3* overexpression lines (*35S::AtDjA3*-8 and -14) were determined by RT-PCR, observing expression of *AtDjA3* gene in all lines analyzed (*OvJ3*-8 and 14, respectively; Supplementary Figure S1D). Several parameters related to the seed morphology such as weight, length, width, and testa structure were analyzed. We observed that *j3* seeds showed a reduction in average seed weight (7.70 mg/500 seeds) in comparison to Col-0 (10.13 mg/500 seeds). With respect to *35S::AtDjA3*-8 and -14 overexpression lines (*OvJ3*-8 and -14), no significant differences in seed weight between overexpression lines and Col-0 were found (**Figure 1A**). In order to evaluate the seed width and length, and testa morphology of the *Atdja3*-null mutant line and *35S::AtDjA3* overexpression lines, micrographs of seeds were taken by eSEM (**Figures 1B–D**, respectively). In agreement with weight data, the *Atdja3*-null mutant seeds are reduced in width in comparison to Col-0 and *35S::AtDjA3* overexpression lines (**Figure 1B**). In addition, the surface of the seed testa in the *j3* mutant line displayed variations in the columella shape compared to Col-0 seeds (**Figure 1D**). With respect to the *35S::AtDjA3* transgenic lines, no significant differences between the overexpression lines and Col-0 were found in seed width and length (**Figures 1B,C**).

### *AtDjA3* Gene is Modulated Under Abiotic Stress

**FIGURE 2:**
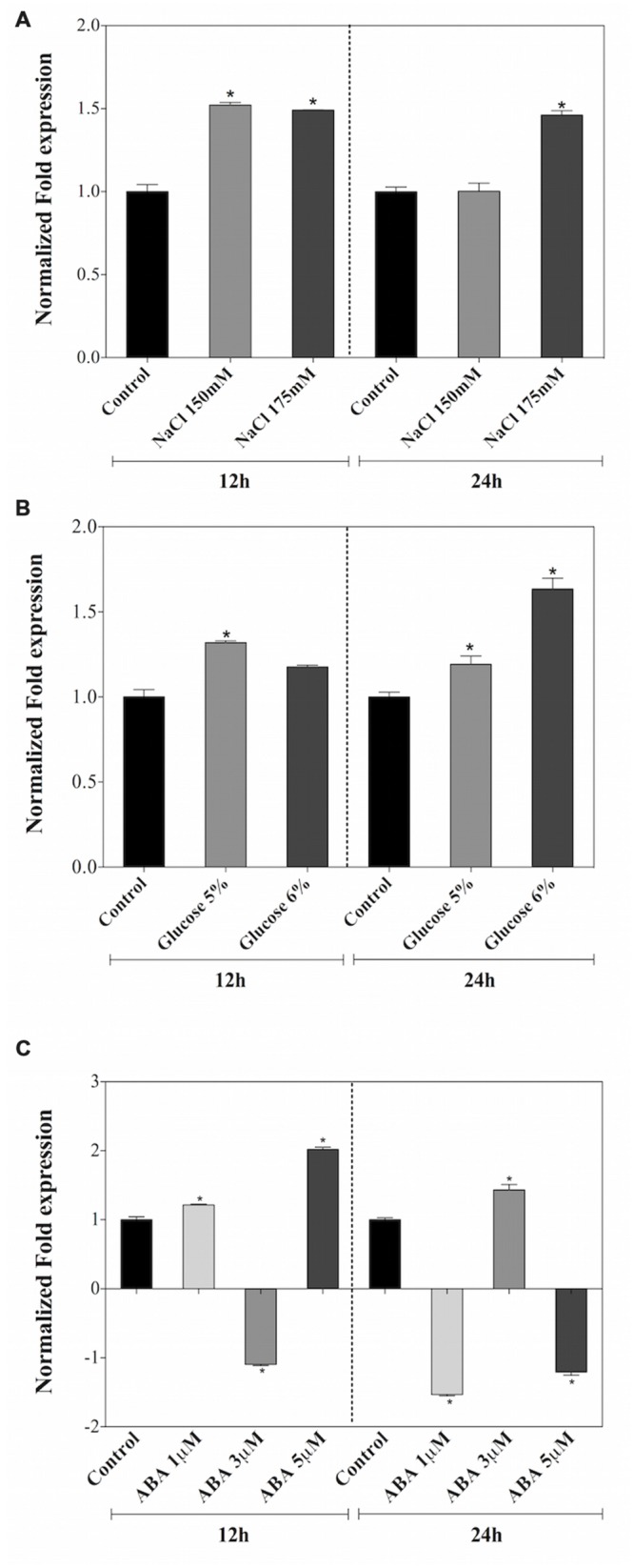
**Expression levels of *AtDjA3* gene under abiotic stress.** The transcript level of *AtDjA3* in *A. thaliana* (Col-0) was determinate in 15-day-old seedlings grown on MS 0.5x liquid medium supplemented with 0, 150, and 175 mM NaCl **(A)**; 0, 5, and 6% glucose **(B)**; 0, 1, 3, and 5 μM ABA **(C)**. Gene expression was determined by qRT-PCR using SYBR green dye. Values represent fold change in expression level upon stressed seedlings compared to non-stressed control seedlings. Quantification was based on a cycle threshold value, with the expression level of the *AtDjA3* normalized to the *Arabidopsis UBQ5* gene. Bars represent mean ± SE (*n* = 3). In case of ratios lower than 1, the inverse of the ratio was estimated and the sign was changed. Asterisks indicate statistically significant differences between the samples treated and untreated, according to the One-way ANOVA analysis and multiple comparison Tukey’s test (*p* ≤ 0.05).

The expression patterns of the *AtDjA3* gene in response to salt and osmotic treatments, as well as the application to ABA hormone were assessed. qRT-PCR experiments were carried out in 15-day-old *A. thaliana* Col-0 seedlings subjected to NaCl (0, 150, and 175 mM), glucose (0, 5, and 6%), and ABA (0, 1, 3, and 5 μM) treatments for 12 and 24 h (**Figure 2**). In both salt treatments, an induction of the *AtDjA3* gene was observed at 12 and 24 h, except for the 24 h 150 mM NaCl treatment (**Figure 2A**). With respect to osmotic stress induction by glucose treatments, a slight expression of *AtDjA3* gene was observed at both treatment times, achieving the highest expression level with 6% glucose at 24 h (**Figure 2B**). Under ABA treatments, increases in *AtDjA3* gene expression were detected with 5 μM of hormone at 12 h, and 3 μM at 24 h (**Figure 2C**). The results showed that *AtDjA3* expression is modulated by abiotic stress.

### The *j3* Mutant Line Showed Less Tolerance to Salt Stress in Germination and Post-germination Stages

**FIGURE 3:**
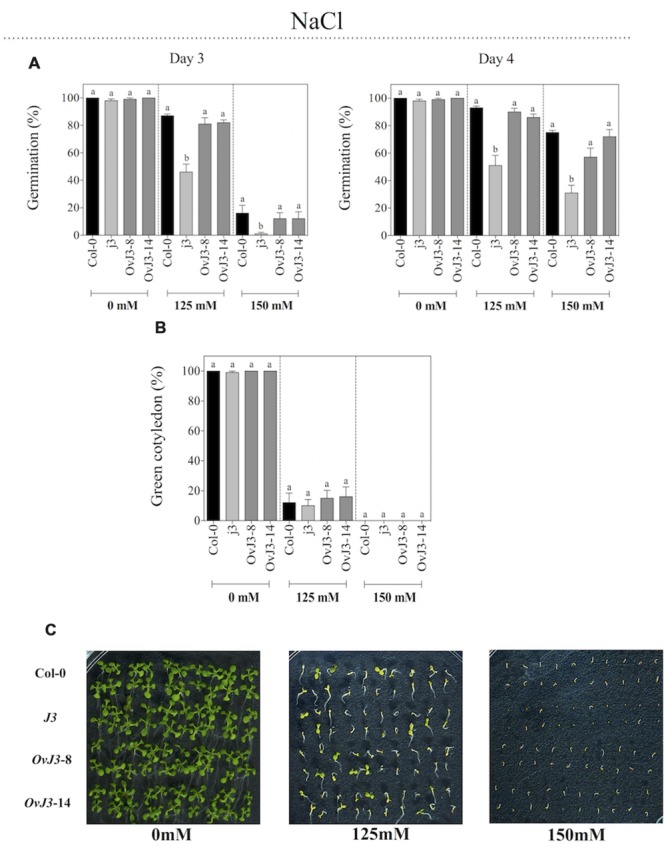
**Germination and green cotyledons development of *Arabidopsis* Col-0, *Atdja3*-null mutant line (*j3*), and *35S::AtDjA3* overexpression lines (*OvJ3*-8 and -14) under salinity.** Seeds were germinated on MS 0.5x (1.5% sucrose) with 0, 125, and 150 mM NaCl. **(A)** Seed germination was evaluated at 3 and 4 days of treatment. **(B)** Percent of green cotyledons were recorded, and the phenotype at 21 days after the stress treatment is presented **(C)**. Bars represent the means ± SE (*n* = 20) of five replicates. Different letters indicate statistically significant differences between the Col-0, *Atdja3-*null mutant line, and *35S::AtDjA3* overexpression lines. One-way ANOVA was used to analyze the differences among treatments were explored through Tukey’s test (*p* ≤ 0.05).

To determine whether *AtDjA3* plays a role in *Arabidopsis* tolerance to salt stress, seeds of Col-0, *Atdja3*-null mutant line (*j3*) and *35S::AtDjA3* overexpression lines (*OvJ3*-8 and -14) were germinated on MS 0.5x medium containing 0, 125, and 150 mM NaCl. We observed that *j3* mutant line was affected during germination under salt treatments in comparison to Col-0 and the *35S::AtDjA3* overexpression lines (**Figure 3A**). At 4 days of 125 and 150 mM NaCl treatments there was a significant decrease in the germination rate of *j3* mutant line (51 and 31%, respectively) compared with Col-0 (93 and 75%, respectively). At 21 days after the germination under salt treatments (0, 125, and 150 mM), the percentage of green cotyledons was recorded (**Figures 3B,C**). At 125 mM NaCl, no significant differences in green cotyledons among the Col-0, *j3* mutant line, and *35S::AtDjA3* overexpression lines were observed (**Figures 3B,C**). Under control conditions, no significant differences in germination rates and green cotyledons among the Col-0, *j3* mutant line, and *35S::AtDjA3* overexpression lines were observed (**Figure 3**).

### The *j3* Mutant Line Showed Increased Sensitivity to Glucose in Germination and Post-germination Growth

**FIGURE 4:**
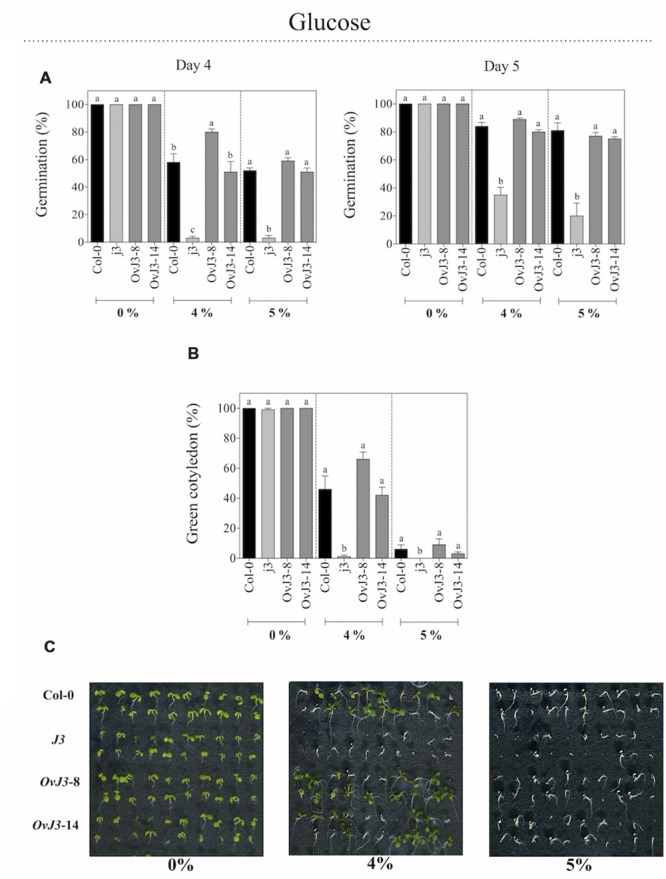
**Germination and green cotyledons development of *Arabidopsis* Col-0, *Atdja3*-null mutant line (*j3*), and *35S::AtDjA3* overexpression lines (*OvJ3*-8 and -14) under osmotic stress.** Seeds were germinated on MS 0.5x (without sucrose) 0, 4, and 5% glucose **(A)** Seed germination was evaluated at 4 and 5 days of treatment. **(B)** Percent of green cotyledons were recorded, and the phenotype at 21 days after the stress treatment is presented **(C)**. Bars represent the means ± SE (*n* = 20) of five replicates. Different letters indicate statistically significant differences between the Col-0, *Atdja3-*null mutant line, and *35S::AtDjA3* overexpression lines. One-way ANOVA was used to analyze the differences among treatments were explored through Tukey’s test (*p* ≤ 0.05).

In addition to salt treatments, we analyzed the germination rates of Col-0, *j3* mutant line, and *35S::AtDjA3* overexpression lines under osmotic stress, by sowing the seeds on MS 0.5x medium containing glucose at 0, 4, and 5% (**Figure 4**). When seeds were sown on glucose, the *j3* mutant line showed a clear reduction in its germination rate with respect to Col-0 and *35S::AtDjA3* overexpression lines (**Figure 4**). At 4 and 5 days, the osmotic sensitivity was noticed in the *j3* mutant line, which achieves only 3% of germination, whereas the Col-0 and overexpression lines exhibited percentages up to 50%. We analyzed the green cotyledons under glucose treatments (**Figures 4B,C**). As shown in germination, the *j3* mutant line showed an arrest in the development of the green cotyledons at both concentrations (**Figure 4C**).

### The *j3* Mutant Line Showed Increased Sensitivity to ABA in Germination and Post-germination Growth

**FIGURE 5:**
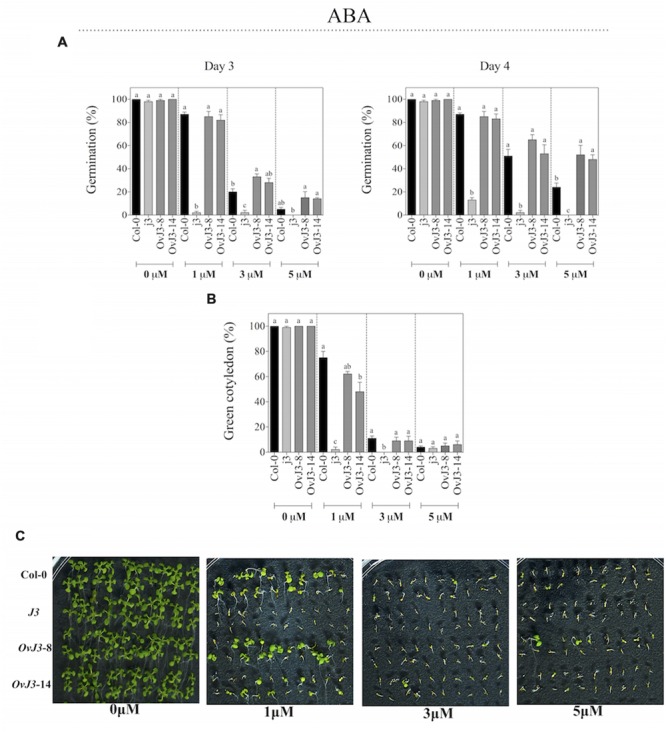
**ABA effect on germination and green cotyledons of *Arabidopsis* Col-0, *Atdja3*-null mutant line (*j3*), and *35S::AtDjA3* overexpression lines (*OvJ3*-8 and -14).** Seeds were germinated on MS 0.5x (1.5% sucrose) with 0, 1, 3, and 5 μM ABA. **(A)** Seed germination was evaluated at 3 and 4 days of treatment. **(B)** Percent of green cotyledons were recorded, and the phenotype at 21 days after the stress treatment is presented **(C)**. Bars represent the means ± SE (*n* = 20) of five replicates. Different letters indicate statistically significant differences between the Col-0, *Atdja3-*null mutant line, and *35S::AtDjA3* overexpression lines. One-way ANOVA was used to analyze the differences among treatments were explored through Tukey’s test (*p* ≤ 0.05).

Inhibitory experiments of seed germination were carried out with Col-0, *j3* mutant line, and *35S::AtDjA3* overexpression lines on MS 0.5x medium containing 0, 1, 3, and 5 μM ABA (**Figure 5**). The *AtDjA3* gene disruption caused a germination sensitivity phenotype on ABA treatments. As observed at 3 and 4 days, the *j3* mutant line exhibited the lowest percentage of germination for all of the ABA concentrations assessed (**Figure 5A**). At the highest concentration of ABA (5 μM), the *j3* mutant line did not germinate, whereas the Col-0 achieved a 24% germination, and the transgenic lines (*35S::AtDjA3*) exhibited more than 40% germination after 4 days (**Figure 5A**). At 21 days after the germination under ABA treatment (0, 1, 3, and 5 μM), the percentage of green cotyledons was recorded (**Figures 5B,C**). The *j3* mutant line exhibits a lower percentage of green cotyledons under ABA treatments. At 1 μM ABA, the *j3* mutant line showed post-germination growth arrest (only 2% of green cotyledons), whereas the Col-0 and the overexpression lines achieved more than 40% of green cotyledons. These data revealed that the *j3* mutant line exhibited an ABA-sensitive phenotype.

### Abscisic Acid-Insensitive 3 (*ABI3*) Gene Expression in the *Atdja3*-Null Mutant Line (*j*3) and *35S::AtDjA3* Overexpression Lines (*OvJ3*) Under ABA Treatment

**FIGURE 6:**
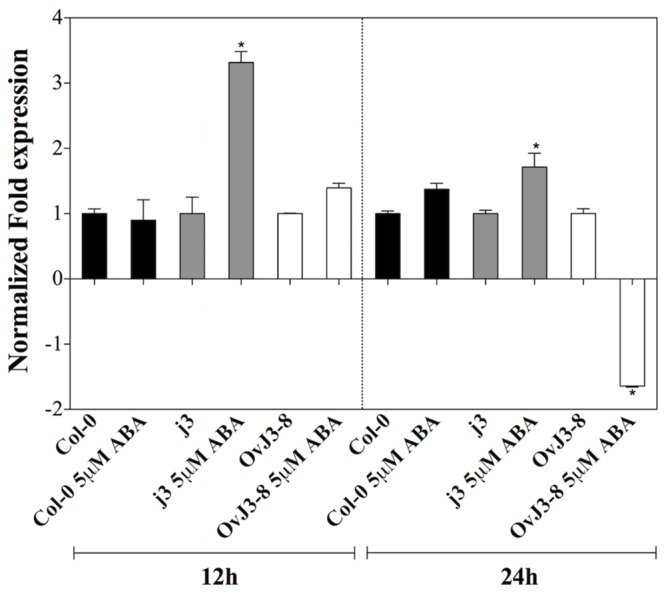
***ABI3* expression levels on *Arabidopsis* Col-0, *Atdja3*-null mutant line (*j3*), and *35S::AtDjA3-8* over-expression line under ABA treatment.** Fifteen-day-old *Arabidopsis* seedlings were placed on MS 0.5x liquid medium supplemented with 0 and 5 μM ABA at 12 and 24 h. The expression levels of *ABI3* were determined by qRT-PCR using SYBR green dye. Data are expressed as relative mRNA level compared to the plants of each line without ABA treatments, and were calculated after normalization to the *Arabidopsis APT1* gene using the comparative threshold method. In case of ratios lower than 1, the inverse of the ratio was estimated and the sign was changed. Bars represent mean ± SE (*n* = 3). Asterisks indicate statistically significant differences between the samples treated and untreated, according to the One-way ANOVA analysis and multiple comparison Tukey’s test (*p* ≤ 0.05).

Based on ABA sensitivity observed in the *j3* mutant line, we examined the expression of *ABI3* transcription factor. For this, qRT-PCR expression analyses were carried out in 15-day-old plants of Col-0, *j3* mutant line, and *35S::AtDjA3-8* overexpression lines by being subjected to ABA treatment (5 μM) for 12 and 24 h (**Figure 6**). The *j3* mutant line showed higher *ABI3* transcript levels under ABA treatment during the entire time course analyzed compared to the parental (Col-0). The highest expression of the *ABI3* gene in the mutant line was observed at 12 h (**Figure 6**). In contrast, the *ABI3* gene was repressed in the *35S::AtDjA3-8* overexpression line at 24 h under ABA treatment.

## Discussion

One of the major molecular mechanisms to re-establish cellular homeostasis and protect cellular components under abiotic stress is the expression of stress response genes, which encode molecular chaperones such as HSP. HSP40, also known as J-proteins, act as molecular chaperones, and are involved in many cellular processes, including development, signal transduction, and resistance to environmental stresses (Rajan and D’Silva, 2009). We have characterized the *Arabidopsis thaliana AtDjA3* gene (At3g44110), which encodes a J-protein, belonging to group I (also known as group A). To determine the possible functional roles of *AtDjA3* in response to abiotic stresses, a T-DNA mutant line and overexpression lines (*35S::AtDjA3*) were obtained and characterized. Remarkably, we found that the *Atdja3*-null mutant line (*j3*) produced smaller and lighter seeds in comparison to parental Col-0. On the other hand, seeds from the overexpression lines showed no significant differences in seed weight or size compared to Col-0 seeds. We noticed that the lack of *AtDjA3* transcript altered the columella shape of the seeds; these alterations could be due to alterations in the shape and size of seed. In agreement with our proposal, the *Arabidopsis* microarray database (*Arabidopsis* eFP Browser^2^) reports that *AtDjA3* gene is induced during stages 8, 9, and 10 of seed development, and also in dry seed (Schmid et al., 2005). Thus, the loss of function of *AtDjA3* gene could be an important factor in seed formation.

We have showed that abiotic stressors modulated the *AtDjA3* gene, including NaCl, glucose, and the application of the ABA hormone. The highest accumulation of *AtDjA3* transcript was observed under ABA treatment, in particular with 5 μM ABA at 12 h. Characterization of the *AtDjA3* gene in the germination process of *Arabidopsis* seeds under abiotic stress conditions using the *j3* mutant line and *35S::AtDjA3* overexpression lines revealed that the loss-of-function of *AtDjA3* resulted in seeds with sensitivity to salt and osmotic stresses. Furthermore, seeds of the *j3* mutant line exhibited increased sensitivity to ABA during germination compared to the parental Col-0. Conversely, *35S::AtDjA3* overexpression lines showed the highest rate of germination after 4 days at the maximum concentration of ABA (5 μM). We also examined the effects of the *j3* mutant line on the post-germination growth in the salt and osmotic stress response. The results showed that cotyledon development in *j3* mutant line was severely inhibited during glucose and ABA treatments, while during salt treatments no differences were observed. On the other hand, the *AtDjA3* overexpression lines showed a similar behavior to parental Col-0 in germination and cotyledon greening under salt and osmotic treatments.

A total of 120 J-domain proteins have been identified in the *Arabidopsis* genome, which represents a large and diverse family of molecular chaperones (Rajan and D’Silva, 2009). Although their functions are mostly uncharacterized, J-proteins have been implicated in plant stress response. In particular, the role of *AtDjA3* in heat and salt stress has been documented. For instance, Li et al. (2007) reported that *AtDjA3* and its paralogous gene, *AtDjA2*, improve *Arabidopsis* thermotolerance. In addition, Yang et al. (2010) showed that plants lacking *AtDjA3* gene are more sensitive to salt at alkaline pH, and exhibit decrease plasma membrane H^+^-ATPase activity. The authors reported that under alkaline conditions, *AtDjA3* interacts with protein kinase 5 (PKS5), repressing PKS5 kinase activity to release plasma membrane H^+^-ATPase. These reports are consistent with our findings, showing that the chaperone AtDjA3 plays a key role during abiotic stress tolerance. Moreover, others J-proteins have been detected during salt and heat stress. For instance, *ANJ1*, a DnaJ gene from *Atriplex nummularia*, was induced by heat and salt stress (Zhu et al., 1993). Similarly, the expression of *SGJ3* (DnaJ-like) was rapidly induced in Japanese willow (*Salix gilgiana* S.) plants upon exposure to heat and salt stress (Futamura et al., 1999). Overexpression of *Arabidopsis DnaJ* gene (type I, At2g22360) in *E. coli* and *Arabidopsis* plants exhibited increased tolerance to NaCl stress (Zhichang et al., 2010).

We noticed that the disruption of *AtDjA3* gene resulted in ABA hypersensitivity. ABA plays an important role in developmental processes such as seed maturation, including synthesis of seed storage proteins and lipids, seed desiccation tolerance, dormancy, control of germination, and the subsequent commitment to seedling growth, and adaptive responses to environmental stimuli in plants (Finkelstein et al., 2002; Cutler et al., 2010; Hubbard et al., 2010; Raghavendra et al., 2010; Fujita et al., 2011). The *j3* mutant line showed sensitivity to ABA in germination and post-germination stages in comparison to parental Col-0. Conversely, *35S::AtDjA3* overexpression lines had less sensitivity to 5 μM ABA in germination than those of Col-0. We evaluated the *ABA-insensitive 3* (*ABI3*) gene expression in the Col-0, *j3* and *35S::AtDjA3* overexpression lines under ABA treatment. We found that both the lack of *AtDjA3* transcript and its constitutive overexpression altered *ABI3* gene expression. *ABI3* expression was induced in the *j3* mutant line in comparison to WT (Col-0) at 12 and 24 h after ABA treatment. This increased induction of *ABI3* transcript in the *j3* mutant line, a key factor in ABA signaling, could be correlated with the ABA hypersensitivity phenotype observed in the *j3* mutant line during germination and post-germination growth. The transcriptional factor ABI3 is considered to be essential for the regulation of seed specific development, so this factor determines the ABA sensitivity and plays a key role in desiccation tolerance and dormancy during zygotic embryogenesis (Zhang et al., 2005). *ABI3* transcript and protein levels are abundant in maturing and mature seeds, but disappear soon after germination. However, these levels can be modulated by ABA or osmotic stress during the time period when post-germination growth arrest occurs (Lopez-Molina et al., 2001, 2002). In contrast with the *j3* mutant, we found that transgenic plants overexpressing *AtDjA3* were slightly more resistant to 5 μM ABA compared to the WT during germination. This could be explained by the down-regulation of the *ABI3* transcript at 5 μM ABA treatment in *35S::AtDjA3* overexpressing line, which is contrasting behavior to that observed in the *Atdja3*-null mutant background.

The results presented here showed that the *j3* mutant line generates smaller seeds, which were more sensitive to abiotic stress and exogenous application of ABA. This phenotype of *j3* mutant line to stress treatments reveals that *AtDjA3* gene might have an important role during the germination process, and provides new insights into abiotic stress responses mediated by chaperones.

## Author Contributions

SS-M, AR-H, MO-A, FS-B designed and carried out the experiments, analyzed the results, and wrote the manuscript. JJ-B designed the research, contributed scientific advice, correction, wrote and revision of the manuscript. All authors have read and approved the final manuscript.

## Conflict of Interest Statement

The authors declare that the research was conducted in the absence of any commercial or financial relationships that could be construed as a potential conflict of interest.

## Footnotes

1 http://signal.salk.edu

2 www.bar.utoronto.ca
