# Supplementary figures and images for: Arabidopsis AtDjA3 Null Mutant Shows Increased Sensitivity to Abscisic Acid, Salt, and Osmotic Stress in Germination and Post-germination Stages
Source: Front Plant Sci. 2016 Feb 25;7:220. doi: 10.3389/fpls.2016.00220 (PMC4766394; doi:10.3389/fpls.2016.00220)

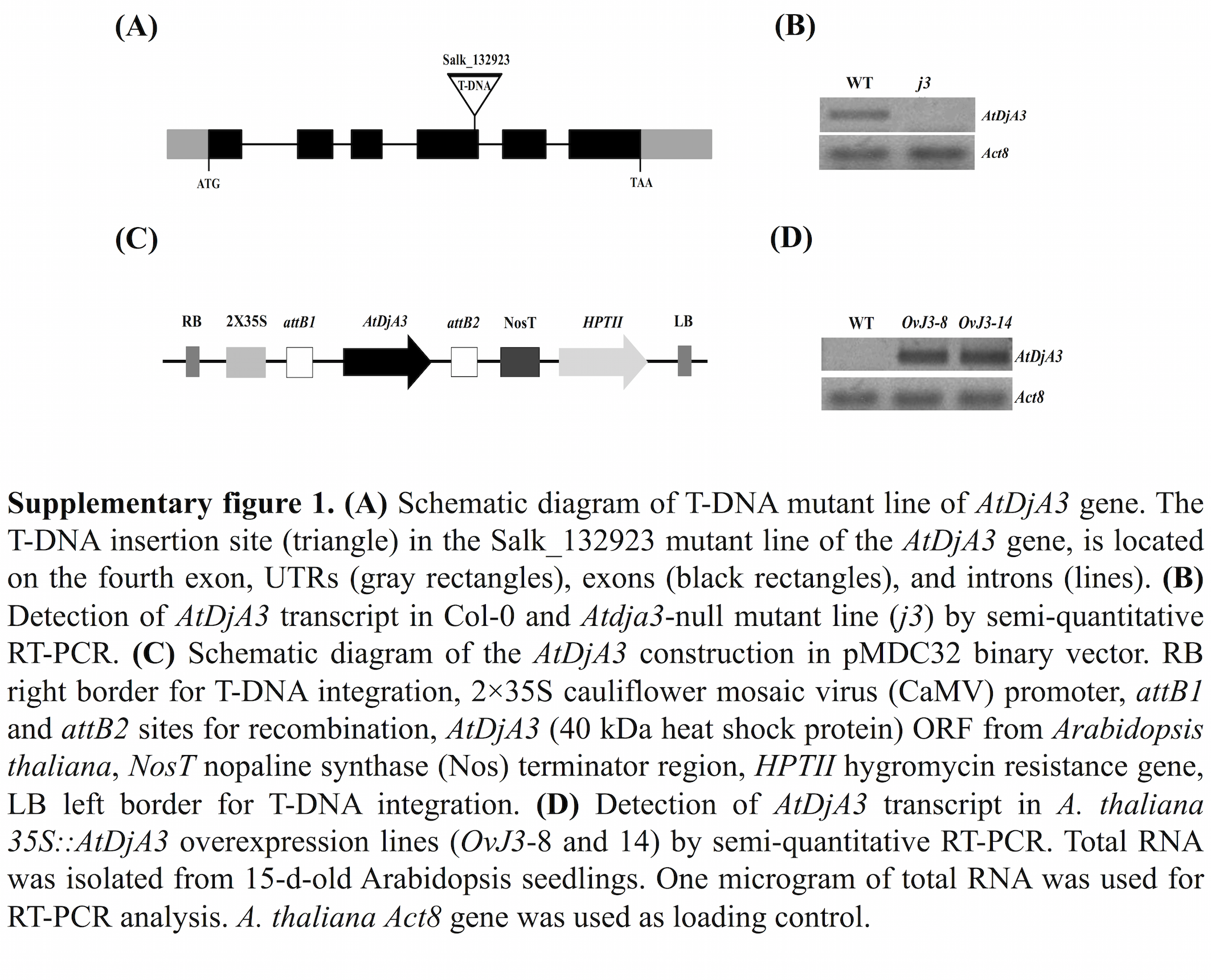

Supplement: Supplementary file 1 [file Image_1.TIFF]
